# Emergence of the B.1.214.2 SARS-CoV-2 lineage with an Omicron-like spike insertion and a unique upper airway immune signature

**DOI:** 10.1186/s12879-024-09967-w

**Published:** 2024-10-10

**Authors:** Andrew Holtz, Johan Van Weyenbergh, Samuel L. Hong, Lize Cuypers, Áine O’Toole, Gytis Dudas, Marco Gerdol, Barney I. Potter, Francine Ntoumi, Claujens Chastel Mfoutou Mapanguy, Bert Vanmechelen, Tony Wawina-Bokalanga, Bram Van Holm, Soraya Maria Menezes, Katja Soubotko, Gijs Van Pottelbergh, Elke Wollants, Pieter Vermeersch, Ann-Sophie Jacob, Brigitte Maes, Dagmar Obbels, Veerle Matheeussen, Geert Martens, Jérémie Gras, Bruno Verhasselt, Wim Laffut, Carl Vael, Truus Goegebuer, Rob van der Kant, Frederic Rousseau, Joost Schymkowitz, Luis Serrano, Javier Delgado, Tom Wenseleers, Vincent Bours, Emmanuel André, Marc A. Suchard, Andrew Rambaut, Simon Dellicour, Piet Maes, Keith Durkin, Guy Baele

**Affiliations:** 1 Lyssavirus Epidemiology and Neuropathology Unit, Institut Pasteur, Université Paris Cité, Paris, France; 2 Department of Microbiology, Immunology and Transplantation, Rega Institute, KU Leuven, Leuven, Belgium; 3 Department of Laboratory Medicine, University Hospitals Leuven, National Reference Centre for Respiratory Pathogens, Leuven, Belgium; 4 Institute of Ecology and Evolution, University of Edinburgh, Edinburgh, UK; 5 Institute of Biotechnology, Life Sciences Center, Vilnius University, Vilnius, Lithuania; 6 Department of Life Sciences, University of Trieste, Trieste, Italy; 7 Fondation Congolaise Pour La Recherche Médicale, Brazzaville, Republic of Congo; 8 Institute for Tropical Medicine, University of Tübingen, Tübingen, Germany; 9 Faculty of Sciences and Techniques, University Marien Ngouabi, Brazzaville, Republic of Congo; 10 General Practice Heiberg, Leuven, Belgium; 11 Department of Public Health and Primary Care, KU Leuven, Leuven, Belgium; 12 Laboratory for Molecular Diagnostics, Jessa Hospital, Hasselt, Belgium; 13 Hasselt University, Hasselt, Belgium; 14 Limburg Clinical Research Center, Hasselt, Belgium; 15 University of Ghent, Ghent, Belgium; 16 Department of Laboratory Medicine, Antwerp University Hospital (UZA), Edegem, Belgium; 17 Laboratory of Medical Biochemistry and Laboratory of Medical Microbiology, University of Antwerp, Wilrijk, Belgium; 18 Department of Laboratory Medicine, AZ Delta General Hospital, Roeselare, Belgium; 19 Institut de Pathologie Et de Génétique, Gosselies, Belgium; 20 Department of Laboratory Medicine, KLINA General Hospital, Brasschaat, AZ Belgium; 21 AZ Sint-Maarten, Mechelen, Belgium; 22 Switch Laboratory, VIB Center for Brain and Disease Research and Department of Cellular and Molecular Medicine, KU Leuven, Leuven, Belgium; 23 Molecular Medicine, KU Leuven, Leuven, Belgium; 24 Center for Genomic Regulation, Barcelona Institute for Science and Technology, 08003 Barcelona, Spain; 25 Universitat Pompeu Fabra, 08002 Barcelona, Spain; 26 Institució Catalana de Recerca I Estudis Avançats, 08010 Barcelona, Spain; 27 Department of Biology, KU Leuven, Leuven, Belgium; 28 Department of Medical Genetics, CHU Liege, Liege, Belgium; 29 Department of Biostatistics, Fielding School of Public Health, University of California, Los Angeles, CA USA; 30 Université Libre de Bruxelles, Brussels, Belgium; 31 Laboratory of Human Genetics, GIGA Research Institute, Liège, Belgium

**Keywords:** SARS-CoV-2, Genomic epidemiology, Phylogeography, Phylodynamics, Disease spread, COVID-19

## Abstract

We investigate the emergence, mutation profile, and dissemination of SARS-CoV-2 lineage B.1.214.2, first identified in Belgium in January 2021. This variant, featuring a 3-amino acid insertion in the spike protein similar to the Omicron variant, was speculated to enhance transmissibility or immune evasion. Initially detected in international travelers, it substantially transmitted in Central Africa, Belgium, Switzerland, and France, peaking in April 2021. Our travel-aware phylogeographic analysis, incorporating travel history, estimated the origin to the Republic of the Congo, with primary European entry through France and Belgium, and multiple smaller introductions during the epidemic. We correlate its spread with human travel patterns and air passenger data. Further, upon reviewing national reports of SARS-CoV-2 outbreaks in Belgian nursing homes, we found this strain caused moderately severe outcomes (8.7% case fatality ratio). A distinct nasopharyngeal immune response was observed in elderly patients, characterized by 80% unique signatures, higher B- and T-cell activation, increased type I IFN signaling, and reduced NK, Th17, and complement system activation, compared to similar outbreaks. This unique immune response may explain the variant's epidemiological behavior and underscores the need for nasal vaccine strategies against emerging variants.

**Supplementary Information:**

The online version contains supplementary material available at 10.1186/s12879-024-09967-w.

## Introduction

Since February 2020, SARS-CoV-2 has rapidly accumulated mutations that have accelerated viral spread. Although its evolutionary rate has been slower than its rate of transmission, SARS-CoV-2 has experienced numerous events of divergent evolution, leading to the emergence of numerous lineages. Some of these lineages and the mutations that define them have been identified as variants of interest (VOI) or variants of concern (VOC). Although the majority of the single nucleotide polymorphisms (SNPs) that characterize these lineages do not result in significant changes in infectivity and virulence, there are key mutations that are associated with an increase in transmissibility or increased host immune escape. These key mutations have led to a rapid viral population replacement in regions where these strains have emerged.

Several mutations have appeared independently in separate VOCs, providing evidence of convergent evolution of mutations that increase the fitness of infection. The D614G mutation in the spike protein, which was absent from the ancestral strain but rapidly became dominant among circulating SARS-CoV-2 lineages, has been found to increase virion spike density and infectivity [1]. Similarly, mutation N501Y has been found to lead to an increased affinity to ACE2, the receptor to which the spike protein binds during host invasion [2]. E484K is a spike substitution that has been associated with circulating variants such as Beta, Gamma, and Omicron lineages [3]. These along with K417N, Y505H, and L452X have been associated with an increased affinity for the virus spike protein to the host ACE2 receptor, which increases the variant's transmissibility and pathogenicity [4–6].

The majority of these mutations of concern have been documented primarily as substitutions, but small spike deletions, such as Δ69/70 and Δ144, have also played a significant role in SARS-CoV-2 evolution [5, 7]. Comparatively, much less attention has been placed on small insertions, even though the spike protein of BA.1, the first Omicron sublineage to spread globally, was characterized by the insertion of the tripeptide Glu-Pro-Glu on position 214 [7]. Building on this, a recent variant of concern, BA.2.86, which raised international alarm in August 2023 for its remarkable number of spike gene mutations and its highly effective immune evasion, contains a four amino acid insertion in a different location in the spike protein [2, 5]. Position 214, found within the N-terminal domain, has been described as a hotspot for recurrent insertions, both prior to and after the advent of Omicron, and was therefore named RIR1 [7]. Despite the identification of several dozen independent insertions at RIR1, only two RIR1-containing SARS-CoV-2 variants besides BA.1 reached significant international spread, i.e. A.2.5 and B.1.214.2. The latter, first identified in Europe in late 2020, circulated for seven months with a two-month peak in April and May of 2021 in Belgium, Switzerland, and France.

The emergence of the B.1.214.2 variant is a part of the larger unfolding of the B.1.214 lineage. The earliest B.1.214 sequences were identified in the Democratic Republic of the Congo in April 2020 [8]. Subsequent sub-lineages were identified (some retroactively post Pangolin definition): B.1.214.2 in Switzerland by late November 2020; B.1.214.1 in the Republic of the Congo in early December 2020; B.1.214.3 by mid-December 2020 in Luxembourg; and B.1.214.4 back in Switzerland by January 2021 [8]. While the pangolin classifications of these variants largely at the time relied on geographic clustering, there are notable mutations that define these lineages [9]. All B.1.214 lineages, including its sub-lineages, carry the mutations I1398V, T1881I, and A4016V in ORF1a. The D614G mutation in the spike gene is not only prevalent in the B.1.214 family but also widespread across many variants such as B.1.17, B.1.351, and B.1.617.2. Uniquely, B.1.214.2, B.1.214.3, and B.1.214.4 share the T716I substitution, while B.1.214.3 further carries T95I and T478K mutations (neutral in terms of in silico predicted stability; -0.21 kcal/mol [10]). In contrast, B.1.214.2 is characterized by mutations Q414K and N450K (neutral in terms of in silico predicted stability, -0.23 kcal/mol and slightly destabilizing, 0.9 kcal/mol, respectively [10]) and the inclusion of the RIR1 insertion sequence (Supp. Figure 2) [9].

Despite Delta replacing most other circulating strains of SARS-CoV-2 in early summer 2021, our group recently demonstrated that co-circulating non-dominant variants of concern (Gamma) and even variants of interest (Mu) were able to cause high-fatality outbreaks in high-risk elderly, even when fully vaccinated [11]. In addition, Delta, Gamma, and Mu nursing home outbreaks revealed a fatal immune signature, characterized by an increase in Th17 activation and high *IFNB1* transcript levels but no difference in type I IFN signalization [11]. The B.1.214.2 variant could have become significant in Europe, as evidenced by its rapid expansion in Belgium. Its origin in Belgium or Europe more broadly remains unknown. Several news organizations prematurely reported on the variant’s origin due to confusion with its parental lineage, B.1.214, which circulated in the Democratic Republic of the Congo [12].

By late February 2021, as the prevalence of this variant increased in Europe, a corresponding rise occurred in the number of B.1.214.2 sequences in Central Africa, where it represented a higher relative frequency among sequenced cases compared to Europe. Between December 2020 and July 2021, B.1.214.2 was identified in 26% of all sequences in the Republic of the Congo, and in March 2021 it accounted for over 50% of the sequenced cases [8]. Therefore, a thorough investigation of the phylogeographic origin and immune characteristics of this variant was warranted.

Here, we investigate the emergence of variant B.1.214.2 using travel history-aware phylogeographic inference to investigate whether cryptic transmission of the variant in Central Africa occurred before its emergence in Europe, or whether B.1.214.2 instead emerged from a circulating sibling strain within Europe. In addition, we perform the first in-depth immunological characterization of this variant by studying a large nursing home outbreak with a moderately high (8.7%) case fatality ratio.

## Methods

### Study design

This investigation was initiated due to a sharp rise in SARS-CoV-2 detections in Belgium, characterized by the presence of a new nine-nucleotide insertion sequence within the spike protein. By March 2021, 211 similar sequences were documented in Belgium, and the Pangolin COVID-19 Lineage Assigner [9] assigned the majority of the sequences to lineage B.1.214, which was most commonly found in the Democratic Republic of the Congo. This lineage was then split into B.1.214.1 and B.1.214.2, the latter being the presently described variant of interest. B.1.214.2 is characterized by the insertion of 9 bp (ACAGATCGA) into the spike protein at position 22,204 (adds the amino acids TDR downstream of R214), as well as the spike amino acid changes Q414K, N450K and T716I. The lineage also carries a 30 bp deletion in ORF3a (25,448–25,478), with approximately half the genomes identified in Belgium also carrying a 9 bp deletion (11,288–11,297) in nsp6, a deletion also observed in the lineages B.1.1.7, B.1.351, B.1.525 and P.1. B.1.214.2 genomes have been deposited in the GISAID database with origins in five additional European countries, North America and Africa.

### Data collection

All SARS-CoV-2 genomes used in this study and corresponding metadata were obtained from GISAID in November 2021, filtering for B.1.214 and derivative sequences from November 2020 to August 2021. Travel history was retrieved when available through the GISAID metadata. Additional metadata was collected by contacting sequencing and test laboratories that documented travel data through contacting the GPs who managed patient care. We collected 14 travel itineraries for their associated sequences. This information can be found in Supp. Table 1.

### Mutation analysis and protein modelling

Modeling of the insertion variants was done over the 6VXX PDB structure by fragment grafting using the *Bridging* command of the ModelX tool suite [13]. Residues V213 and L219 provided the anchoring geometries to search for 7-length compatible peptide fragments, resulting in an insertion of 3 residues. The command grafted all geometrically compatible fragments in the ModelX database and obtained models were renumbered to accommodate the insertions corresponding to both B.1.214.2 and A.2.5 variants. Fragment search was blind to the sequence so the side chains of the variants were modeled on all the results obtained using the *BuildModel* command of the FoldX package [14], Post election of the energetically more favorable states was done according to total stability energy calculated with the FoldX *Stability* command.

Proteins lacking a crystal structure were modeled using I-TASSER [15]. Models were visualized using YASARA [16], and schematic representations of proteins were generated using Protter [17]. FoldX version 3.0 beta 6 [18] was used to predict the effect of the mutations on the thermodynamic stability and the interaction energy. Crystal structures were repaired using the *RepairPDB* command, mutations were modeled using the *BuildModel* command, and interaction energy was analyzed using the *AnalyseComplex* command.

### Phylogenetic analysis

We downloaded all B.1.214.2 (*n* = 1587) and B.1.214-derived sequences (B.1.214*) (*n* = 399) available on GISAID on October 19, 2021, resulting in a total of 1986 sequences. From this preliminary dataset, we used NextClade v1.7.1 [19] to identify sequences of poor quality and Pangolin v1.2.81 [9] to characterize the sequence lineage, which filtered 259 sequences, resulting in 1727 high-quality sequences. The sequences removed were well spread over the range of countries represented, and no countries were eliminated due to sequence quality. We then added these sequences to a NextStrain [20] build of Central African Sequences and global sequences between 28 December 2019 and 08 July 2021, which is the last date of sampling for a B.1.214 divergent sequence. The final dataset contained 3507 sequences and the phylogenetic tree was analyzed using NextStrain and visualized by Auspice [20]. We time-calibrated and rooted the tree using TreeTime v.0.8.6 [21] and “hCoV-10/Wuhan/Hu-1/2019” (GenBank accession MN908947) as the outgroup. We detected and removed 65 outliers. We then extracted the subtree containing all 1662 sequences clustering under the B.1.214* node for further analysis.

We performed maximum likelihood phylogenetic tree reconstruction using IQTREE2 v2.2.2.2 [22] on the sequences found within this B.1.214* cluster. The reconstruction was based on a GTR model with empirical frequencies and a three-category FreeRate model of site heterogeneity. This model was selected as the best-fitting model using IQTREE's ModelTest functionality. To ensure thorough exploration, we conducted a robust search by considering all possible nearest neighbor interchanges (NNIs). Additionally, we optimized the number of initial parsimony trees with a maximum likelihood (ML) NNI search, setting the value to 100. We evaluated the temporal signal of our dataset using TempEst [23], and used TreeTime v0.8.6 [21] to root the tree on divergent B.1.214 sequences and use it as a starting tree for our Bayesian analyses, setting the clock rate to 0.0008, according to the Nextstrain [20] parameter. No outliers were removed since we had already performed outlier removal in the previous dating step.

To determine the molecular clock that best fits the data, we employed the Bayesian Evaluation of Temporal Signal (BETS) [24] analysis in BEAST v1.10.5 [25]. This utilizes Bayes factors to objectively assess the presence of temporal signal in a dataset and determine the feasibility of calibrating a molecular clock (strict vs relaxed) using the associated sampling dates of genetic sequences. The evaluation is done by comparing the ratio of marginal likelihoods between competing models: a heterochronous model that uses the sequences’ sampling dates, and an isochronous model, where all the sampling dates have been removed. We performed four parallel analyses using a non-parametric coalescent prior and a HKY + Γ nucleotide substitution model due to unequal transition rates: 1) strict clock without dates, 2) strict clock with dates, 3) relaxed clock without dates, 4) relaxed clock with dates. We ran the MCMC chains for 10^8 states and used generalized stepping-stone sampling [26] with 100 path steps of 10^6 iterations to compute the marginal likelihoods. The relaxed clock with dates yielded the highest log marginal likelihood and was therefore selected for subsequent analyses.

### Travel history-aware phylogeographic reconstruction

As our main goal was to estimate the origin and understand the historical transmission of B.1.214.2, we selected a subtree containing sequences that clustered with B.1.214.2, yielding 1346 sequences including five pangolin-defined B.1.214 sequences that cluster with B.1.214.2 (bold are shown in Fig. 3a: EPI_ISL_1524721, EPI_ISL_1661248, EPI_ISL_1854777, EPI_ISL_2788222, EPI_ISL_4096556). Although 48% of the sequences were collected in Belgium, we decided not to subsample the dataset in order to not add an additional intervention that could be biased. In the event that Belgium overrepresented the phylogeographic result, we could subsample. This was, however, not the case.

To perform the travel history-aware discrete phylogeographic analysis, we needed to prepare the travel history data. Eleven out of 14 sequences with acquired travel history were collected in Belgium. Each country of origin and country of travel was noted, as well as the sampling date. The days between trip departure and sample collection were fixed if known to estimate the potential infection time. For sequences without date of travel information, we used a random time calculated from a normal prior distribution with a mean of 10 days before the sampling date and a standard deviation of 3 days. This is dictated in the travel history-aware phylogeographic analysis protocol [27, 28].

We performed the travel history-aware Bayesian analysis using BEAST v1.10.5 [25] (pre_thorney_0.1.2). We used the general time-reversible substitution model with estimated base frequencies, gamma site heterogeneity model, and 4 gamma categories. To infer ancestral locations, we used a generalized linear model on the log phylogeographic transition rates [29], using three different covariates- binary neighbor sharing (1—two countries share a border, 0- two countries do not share a border), geographic distance (km) between capital cities, and finally the number of flights between two countries between 12–2019 to 07–2021 provided by Bluedot [30]. Since Liechtenstein does not have its own airport, these sequences were relabeled as Switzerland. We also estimate state change counts by calculating the number of expected transitions between two states (countries) called Markov jumps [31] in the dataset. We used an uncorrelated relaxed clock log-normal relaxed distribution and a non-parametric skygrid coalescent model with the population size as 50 and cut-off of 1.7. skygrid’s parameters were inferred by Hamiltonian Monte Carlo sampling [32].

Each Markov chain ran for 2 × 10^8 states and was sampled every 100,000th state. Convergence was confirmed in Tracer v1.7.1 [33] by reviewing effective sample sizes (ESSs). All chains were combined using Logcombiner, giving a final MCMC length of 1.5 × 10^12^ states.

To adequately approximate the posterior distribution, the MCMC was run for 1.5 × 10^12 states, sampling every one million states. Convergence and mixing was confirmed in Tracer v1.7.1 [33] once effective sample sizes (ESSs) reached 200 for every parameter. We summarized all trees by constructing a maximum clade credibility (MCC) tree using TeeAnnotator. Branch heights were summarized by the common-ancestor model, which summarizes branch heights of clades across all posterior trees and not only the values for a subset of trees that have that clade [34].

### ACE2 binding *pseudo*-neutralization assays

Pseudoneutralization assays, consisting of competitive binding between antibodies and soluble ACE2 receptor to plate-coated Spike proteins of 10 different VOC (MSD), was performed and cross-VOC neutralization calculated as previously described [35].

### Whole genome sequencing

Positive SARS-CoV-2 samples with a sufficiently high viral load (> 1000 RNA copies/ml) were analyzed at the Rega Institute for Medical Research at KU Leuven using whole-genome sequencing, as part of a larger consortium of sequencing laboratories throughout Belgium (this totaled over 12,000 samples between January 1, 2020, and July 31, 2021). An automated RNA extraction was performed using the MagMAx Viral / Pathogen kit II (MVPII) (Thermo Fisher Scientific, A48383) with 200 μl sample input. The genomes were amplified following the ARTIC network protocol V3 [36] or as described by Freed et al*.* [37]. After clean-up of the amplicons, libraries were prepared using the SQK-LSK109 + EXP-NBD196 ligation sequencing kit from Oxford Nanopore Technologies. Subsequently, the libraries were quantified, and sequencing was performed on a GridION platform using MinKNOW’s built-in base calling, demultiplexing, and adapter trimming. Sequencing runs were processed using the ARTIC analysis pipeline and custom scripts.

### Digital transcriptomics analysis of upper airway samples

Comprehensive immune profiling of upper airway samples was performed by 600-plex targeted analysis by digital nCounter transcriptomics (NanoString) in a subset of residents with sufficient leftover diagnostic samples (*n* = 17, of which 13 were PCR-positive). RNA was extracted from nasopharyngeal swabs as described above for whole genome sequencing and used for hybridization to pre-specified Human Immunology V2 and customized SARS-CoV-2 panels, as described previously [38–40]. Pathway score analyses and cell type deconvolution were performed using nSolver software (NanoString Technologies Ltd.).

## Results

### Detection and Genomic Surveillance of SARS-CoV-2 lineage B.1.214.2

Between January 3 and January 19, 2021, seven patients were identified in Belgium who had recently returned from trips to the Republic of the Congo (GISAID sequence IDs: EPI_ISL_890291, EPI_ISL_890294, EPI_ISL_833185), Kinshasa, Democratic Republic of the Congo (EPI_ISL_912424, EPI_ISL_1123370), and Andalusia, Spain (EPI_ISL_894200, EPI_ISL_894201). These seven samples were not unique in their clinical presentation, but they all tested positive for a unique strain of SARS-CoV-2, which had a characteristic set of mutations—most notably a three amino acid (AA) insertion sequence at position R214 in the spike (S) protein. Instances of this unique variant rose in the following weeks, ultimately leading to the Pangolin definition of the new lineage, B.1.214.2, on March 02, 2021.

**Fig. 1:**
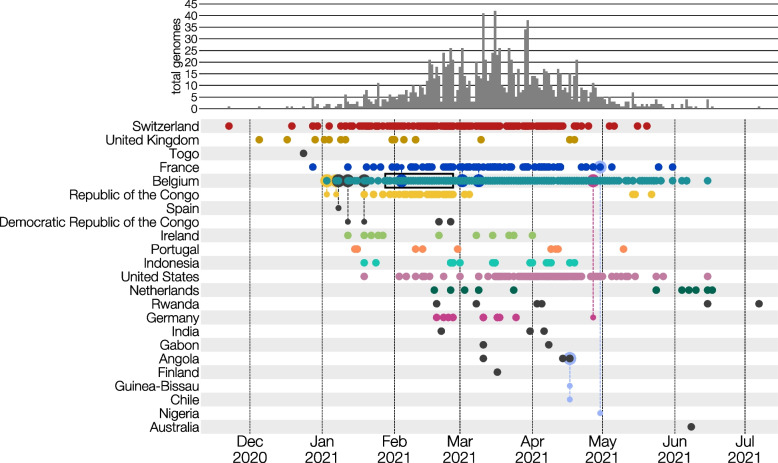
International dispersal of SARS-CoV-2 lineage B.1.214.2. Dots represent sequenced cases deposited to GISAID from November 2020 to July 2021. The majority of sequenced cases were collected between February and June 2021. Colored countries represent countries with ten or more sequences. Dotted lines between dots represent a travel-history included in the analysis. Countries that are included only by the result of travel-history interviews are colored light blue. The overall accumulation of genomes from the time period is represented above by a bar chart showing total B.1.2142 genomes by week. The time range of the nursing home outbreak in Belgium is indicated by a black box

Upon definition, the first case of B.1.214.2 in GISAID was identified to be from Switzerland, collected on November 11, 2020 (EPI_ISL_1296843; Fig. 1). The lineage quickly rose and declined between November 2020 and July 2021 with a peak in March and April 2021 (Fig. 1). During this period, 1587 B.1.214.2 genomes were deposited to GISAID from a small set of countries- the top six most represented being Belgium (742, 46.8%), Switzerland (266, 16.8%), France (231, 14.6%), USA (126, 7.9%), Republic of the Congo (49, 3.1%), and Indonesia (29, 1.8%). Of the twenty countries presented, six of them are from Central Africa (the Republic of the Congo, the Democratic Republic of the Congo, Angola, Rwanda, and Gabon; Fig. 1 and Supp. Figure 1).

**Fig. 2:**
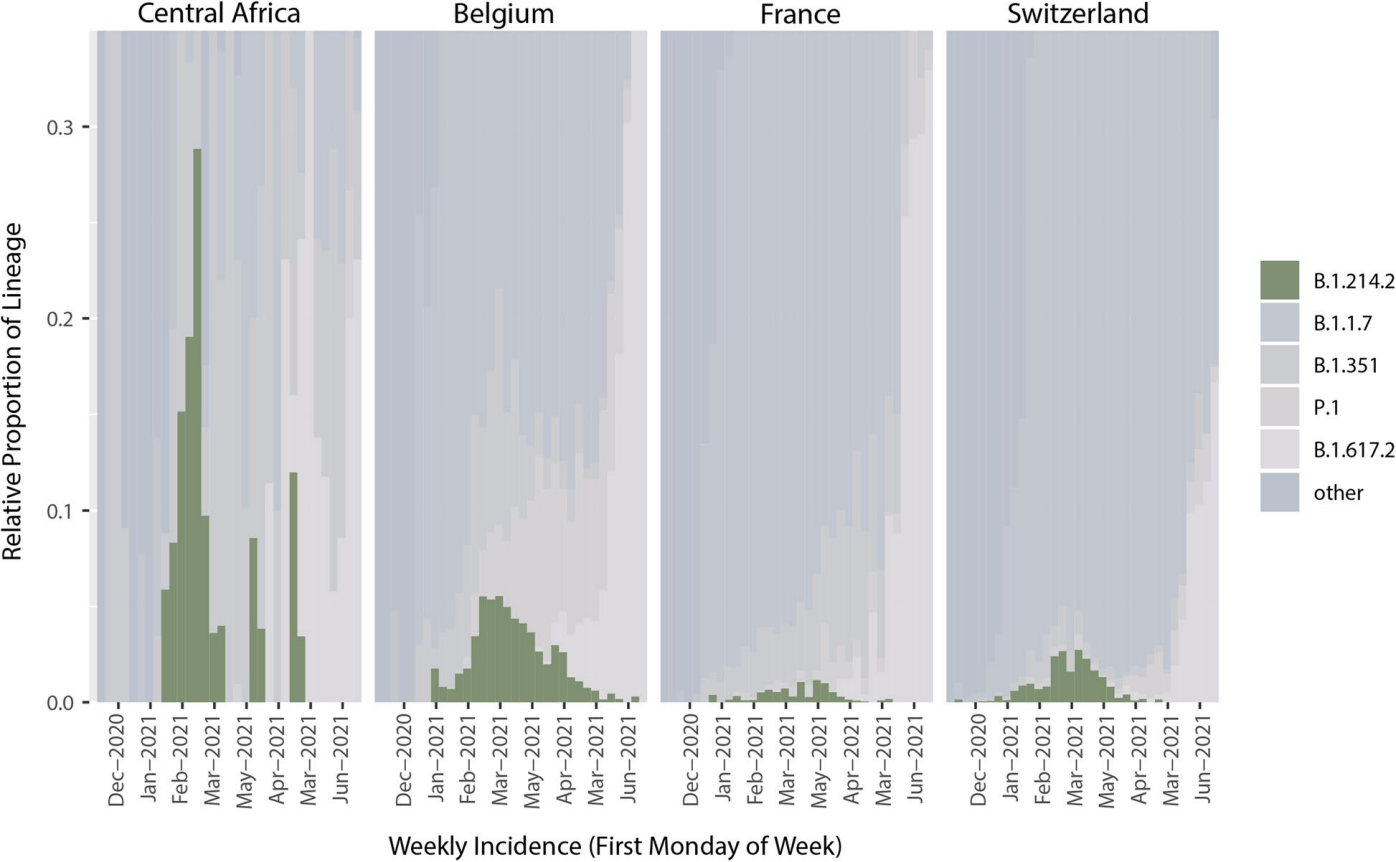
Relative proportions of SARS-CoV-2 lineages from GISAID in four countries (Central Africa, Belgium, France, Switzerland). Due to a limited number of sequences, the Republic of the Congo, the Democratic Republic of the Congo, Angola, and Gabon are aggregated into one ‘Central Africa’ definition. The dark green color represents B.1.214.2 sequences. A high proportion of B.1.214.2 sequences is visible in Central Africa during January and February 2021. Peaks in Europe occurred in February and March 2021. The prevalence is clearer in Belgium and in Switzerland than in France due to the presence of other lineages at the time in France

**Fig. 3:**
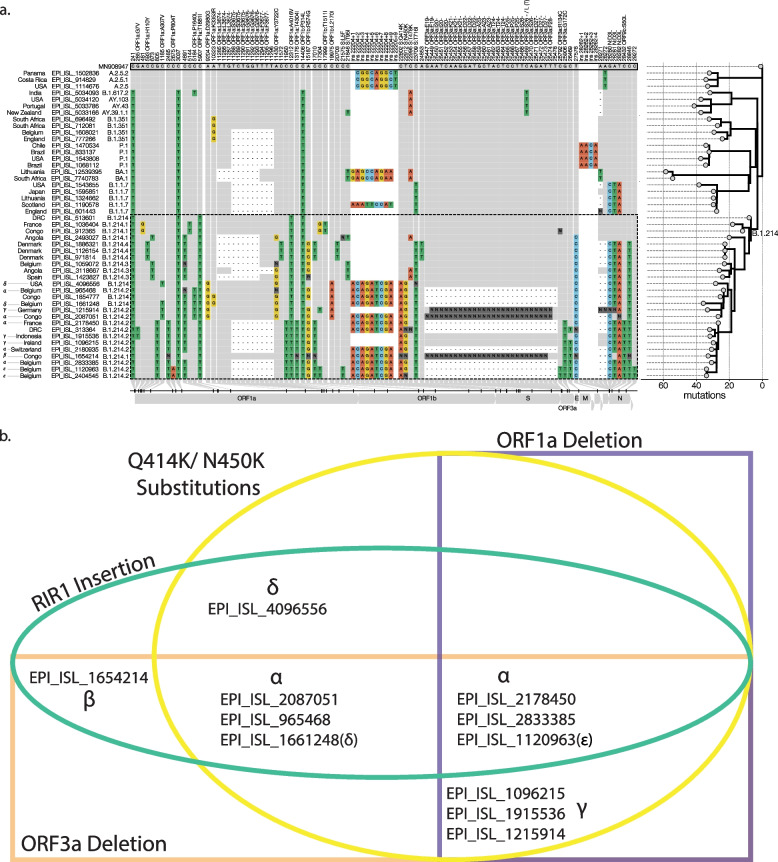
Lineage-defining SNPs and insertion of lineage B.1.214.2. **a** Genomes belonging to the B.1.214 clade are defined inside the dashed-lined box. SNPs that differentiate from the reference (GenBank accession MN908947) and are found in at least two B.1.214 sequences are shown in the condensed SNP alignment. Nucleotides that are shared with the reference strain are shown in grey, while changes from the reference are colored and ambiguities are shown in dark grey. The phylogeny (branch lengths in the number of mutations) on the right shows the relationships between depicted genomes and was rooted on the reference sequence. The long branch defining B.214 is labeled as ‘B.1.214’. Greek letters represent groups of sequences with note-worthy mutational profiles. α = pangolin definition B.1.214.2 (Q414K, N450K, Insertion, and orf1a (50%) and orf3a deletions), β = B.1.214.1 with insertion sequence but missing critical Q414K and N450K, δ = B.1.214 with insertion, Q414K, N450K- possible misclassification by pangolin, ε = Belgian nursing home outbreak; γ = B.1.214.2 that lack insertion sequence but contain Q414K, N450K. **b** Venn diagram separated by different lineage-defining mutations. Greek letters represent the same sequences as described above. α represents B.1.214.2 both with and without the orf1a deletion

The variant's prevalence was far from negligible, peaking at different times across regions: 28.85% in Central Africa (Feb 2, 2021), 5.54% in Belgium (Feb 28, 2021), 2.73% in Switzerland (Mar 7, 2021), and 1.11% in France (Mar 14, 2021) (Fig. 2). transmission of B.1.214.2 began in early 2021, expanding rapidly in March and April. By end-May 2021, its detection declined sharply, largely replaced by other variants, including the Delta variant (B.1.617.2) (Figs. 2 and 3). Adjusting for population, B.1.214.2 was more prevalent in cosmopolitan areas like Brussels, Île-de-France, and Basel, independent of total sequenced cases (Supp. Figure 3). Specifically, 33.6% of Belgian, 48.1% of French, and 69.2% of Swiss B.1.214.2 cases were from these regions, respectively. These areas, known for high visitor and foreign worker/resident numbers, showed higher prevalence despite similar sequencing rates to other regions (Supp. Figure 4), suggesting factors other than sequencing capacity influenced its spread. For instance, Zürich, with minimal border proximity, had only 1.5% of Swiss B.1.214.2 cases, indicating a cross-border transmission effect. By late May 2021, B.1.214.2 incidence nearly vanished in these countries.

### B.1.214.2 Mutation Profile, spike protein carries a novel 3 AA insertion sequence and numerous substitutions also found in variants of concern

The unique 3 AA insertion sequence of lineage B.1.214.2 first caught our attention and led us to investigate the lineage and its introduction to Belgium further. At the time, it was the first variant to have an insertion sequence mutation in the spike protein, and before official Pangolin classification, many sequencing labs did not use technology that was insertion aware and insertions were removed as possible errors. Quite substantially, we found 174 B.1.214.2 sequences from Switzerland that lacked the 3AA insertion sequence. These samples originated from a lab at ETH Zurich which used a V-pipe configuration that was not insertion-aware at the time.

The pangolin definition of B.1.214.2 is characterized by the insertion of 9 bp (ACAGATCGA) into the spike protein at position 22,204 (adds the amino acids TDR downstream of R214), as well as the spike amino acid changes Q414K, N450K, and T716I. The lineage also carries a 30 bp deletion in ORF3a (25,448–25,478), with approximately half the genomes identified in Belgium (or 75% of all B.1.214.2) carrying a 9 bp deletion (11,288–11,297) in ORF1a (Fig. 3a). The strongest indicators of a sequence clustering with B.1.214.2 sequences are the Q414K and N450K substitutions (Fig. 3b, α). In fact, there are four B.1.214.1 sequences from the Republic of the Congo in February 2021, EPI_ISL_1654212, EPI_ISL_1654213, EPI_ISL_1654214, and EPI_ISL_1671927 (Fig. 3b, β), which contain the TDR insertion and the T716I substitution, but lack the critical defining mutations Q414K and N450K. In contrast, there are a number of B.1.214.2 sequences that contain Q414K and N450K but lack the TDR insertion such as EPI_ISL_1096215 (Ireland), EPI_ISL_1915536 (Indonesia), EPI_ISL_1215914 (Germany) (Fig. 3b, γ). Lastly, there are sequences characterized as B.1.214 which contain the Q414K, N450K, and the TDR Insertion, EPI_ISL_4096556 (Fig. 3a, δ), EPI_ISL_1661248, EPI_ISL_1854777, which cluster with B.1.214.2 on the phylogenetic tree, suggesting a discrepancy with Pangolin. The presence of T716I and absence of Q414K and N450K in an earlier B.1.214 sequence from the Republic of the Congo (EPI_ISL_1654214) suggests that the insertion sequence could have been found in ancestors of B.1.214.2, and brought stability to the spike protein to allow for more virulent mutations such as first T716I, and then Q414K and N450K, bringing rise to the B.1.214.2 clade. Interestingly, the ORF1a deletion has occurred independently in all VOC lineages, with the exception of Delta [41]. The presence of the ORF3a deletion in almost all the B.1.214.2 sequences indicates a vital role in clade definition, in contrast to ORF1a, which is only present in less than half of the B.1.214.2 genomes. In fact, several sequences from the Democratic Republic of the Congo (such as EPI_ISL_3133642) have the ORF1a deletion and are estimated in the phylogenetic tree (Fig. 5a) as descendants of the European clades, indicating that transmissions from Europe to Central Africa also occur.

### B.1.214.2 mutations likely to have substantial structural effects on viral function

**Fig. 4:**
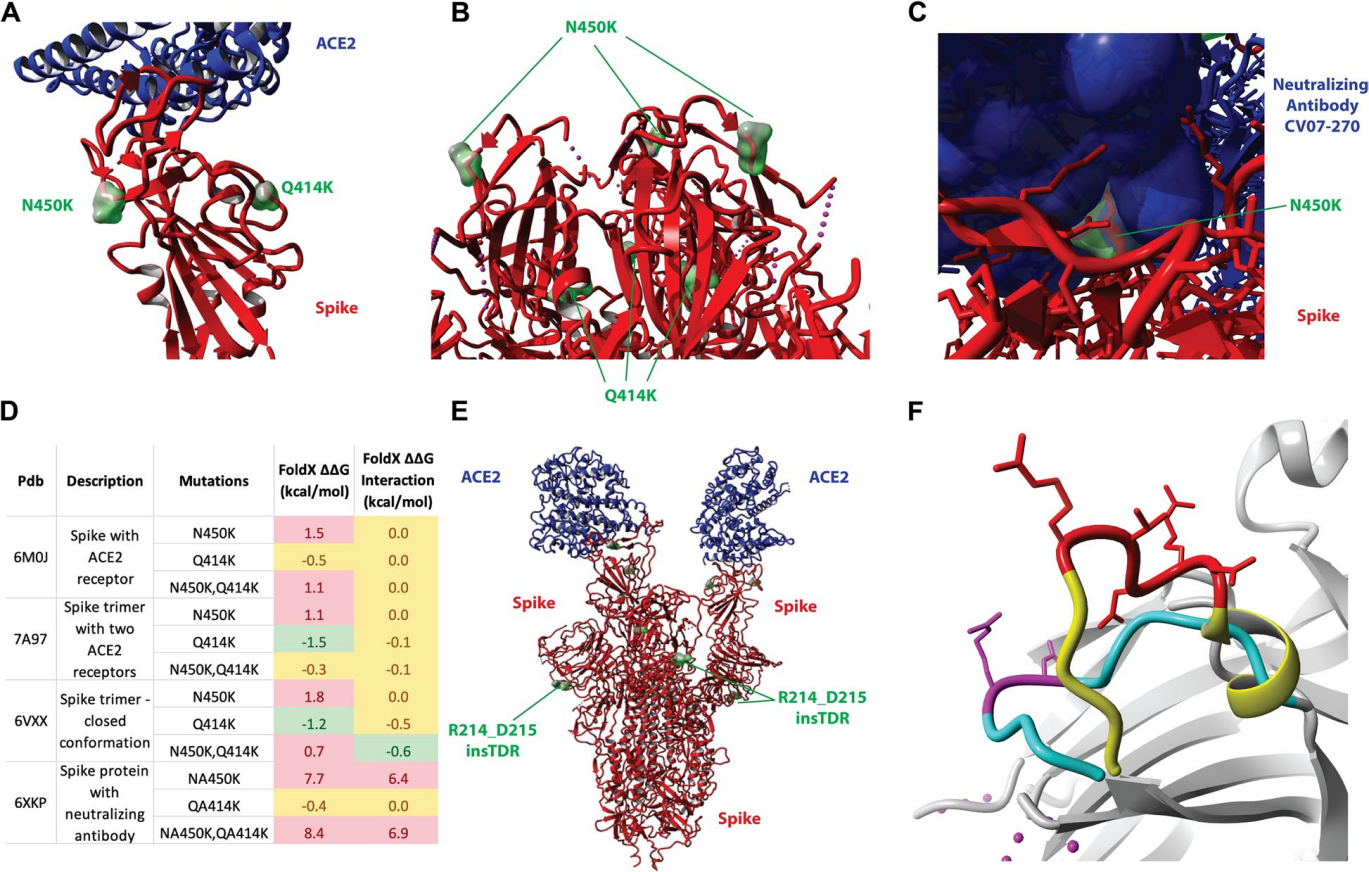
Structural changes of B.1.214.2 variant in the Spike protein and their functional effects on ACE2 receptor and neutralizing antibodies. **A** Spike protein in open conformation bound to ACE2. PDB ID: 6VXX (https://doi.org/10.1016/j.cell.2020.02.058). Red: Spike protein; blue: ACE2 receptor; green: mutations site. **B** Spike protein trimer in closed conformation (not bound to ACE2). PDB ID: 6M0J (https://doi.org/10.1038/s41586-020-2180-5). Red: Spike protein; blue: ACE2 receptor; green: mutations site. **C** The N450K mutation. Crystal structure 6XKP (https://doi.org/10.1016/j.cell.2020.09.049). Blue: antibody; red: spike protein; green: N450. **D** Effect of mutations on thermodynamic stability and interaction energy predicted by FoldX for different types of complexes. Values are in kcal/mol, above 0.5 is deemed destabilizing and below -0.5 is deemed stabilizing, in between -0.5 and 0.5 is considered to have no effect. **E** Structure of the complex between the Spike protein and the ACE2 receptor (PDBid: 7A97 (https://doi.org/10.1038/s41586-020-2772-0)). Red: spike protein; blue: ACE2 receptor; reen: insertions site. Modeling of the effect of Belgian spike insertion compared to WT spike protein. **F** The structure of the wildtype loop is shown in cyan, and the insertion site is highlighted in dark blue. The structure of the modeled Belgian loop is shown in orange, and the insertion is highlighted in red

The N450K mutation is located on the exterior of the Spike protein at a location where immune-evading mutations are often found [6] (Fig. 4A-C). This mutation is predicted to destabilize the spike protein slightly, which is compensated by the Q414K mutation, which is located on the interior of the Spike protein and stabilizes the structure thermodynamically (Fig. 4A). Studies have found that N450K confers a mild increase in ACE2 binding affinity [42].

The N450K is often found at the interface between neutralizing antibodies and the Spike protein (Fig. 4C). The mutation is predicted to decrease the affinity of neutralizing antibodies to Spike (Fig. 4D). Mutating N450K is predicted by FoldX to destabilize the spike protein by more than 0.8 kcal/mol, which is considered destabilizing, for both the closed and open/ACE2-bound conformation (FoldX ΔΔG), while not affecting the interaction energy with ACE2 (FoldX Interaction ΔΔG). Mutating N450K in an example structure of the Spike protein bound to a neutralizing antibody was predicted to severely destabilize the interaction energy, suggesting immune evasion for that particular neutralizing antibody. Several other neutralizing antibodies were predicted to lose their neutralizing effect upon mutating N450K (results not shown). Additionally, the Q414K mutation is exposed in the open conformation of the Spike protein, when it is bound to the ACE2 receptor (Fig. 4A).

The three-amino acid insertion encompasses the sequence TDR between R214 and D215 (Figs. 4E and F). We modeled using ModelX [13] the conformation of the insertion to the exterior of the spike protein in both the open and closed conformation, which is not the interaction site with ACE2 (Figs. 4E and F).

### Phylogeographic analyses reveal the Republic of the Congo as the likely origin of B.1.214.2

We conducted maximum likelihood (ML) phylogenetic and travel history-aware Bayesian phylogeographic analyses with 14 identified travel cases to elucidate the global diffusion of B.1.214.2 and estimate its ancestral origin (Supp. Table 1). The initial ML phylogenetic tree was reconstructed using IQTREE2 v2.2.2.2 [22] with 1662 B.1.214 and descendant sequences (B.1.214, B.1.214.1, B.1.214.2, B.1.214.3, B.1.214.4). A root-to-tip regression analysis showed sufficient temporal signal for a time-scaled phylogenetic inference. We generated such a phylogenetic tree with a calibrated time scale using TreeTime v.0.8.6 [21], dating its most recent common ancestor (tMRCA) to February 2020, indicating the time of divergence of this clade and its derivatives. We were interested specifically in the origin of the B.1.214.2 clade and continued with its subtree for the remainder of the analysis.

**Fig. 5:**
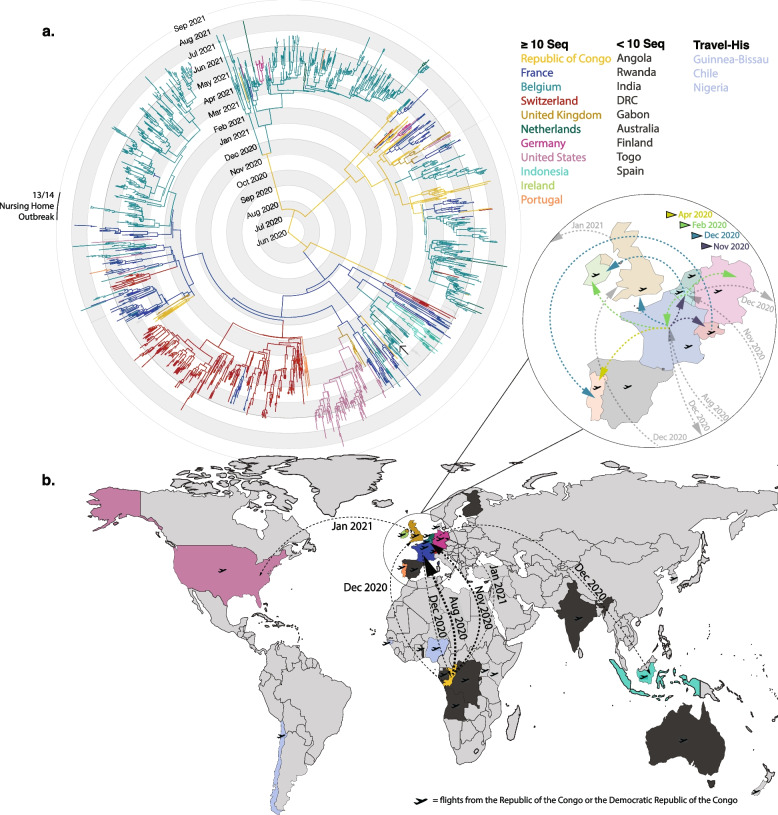
Geographic spread of SARS-CoV-2 lineage B.1.214.2. **a** MCC tree from travel history-aware phylogeographic analysis presenting ancestral country estimations of the B.1.214.2 variant. Colored branches and tips indicate the country of branch origin. Timescale is displayed radially as month and year starting from June 2020. Countries with less than 10 sequences are shown in dark grey. Countries added to the analysis from travel histories are presented in light blue. Tips in the tree that represent sequences from the nursing home outbreak in Belgium are indicated. The solo arrow indicates one nursing-home sequence which is separate from the rest **b**) World map displaying the countries with B.1.214.2 cases. Colors follow the same as above, while light grey countries are absent of B.1.214.2 cases. The plane icon next to the country indicates inbound flights from the Republic of the Congo (RC) and the Democratic Republic of the Congo (DRC). Arrows show the first two introductions to each country or transmissions that result in 15 or more tips from the MCC tree. The date of introduction is shown along the arrow. Specifics are shown in Supplementary Table 2. Liechtenstein was compiled with Switzerland and Luxembourg compiled with Belgium, as sequencing was performed across borders

**Fig. 6:**
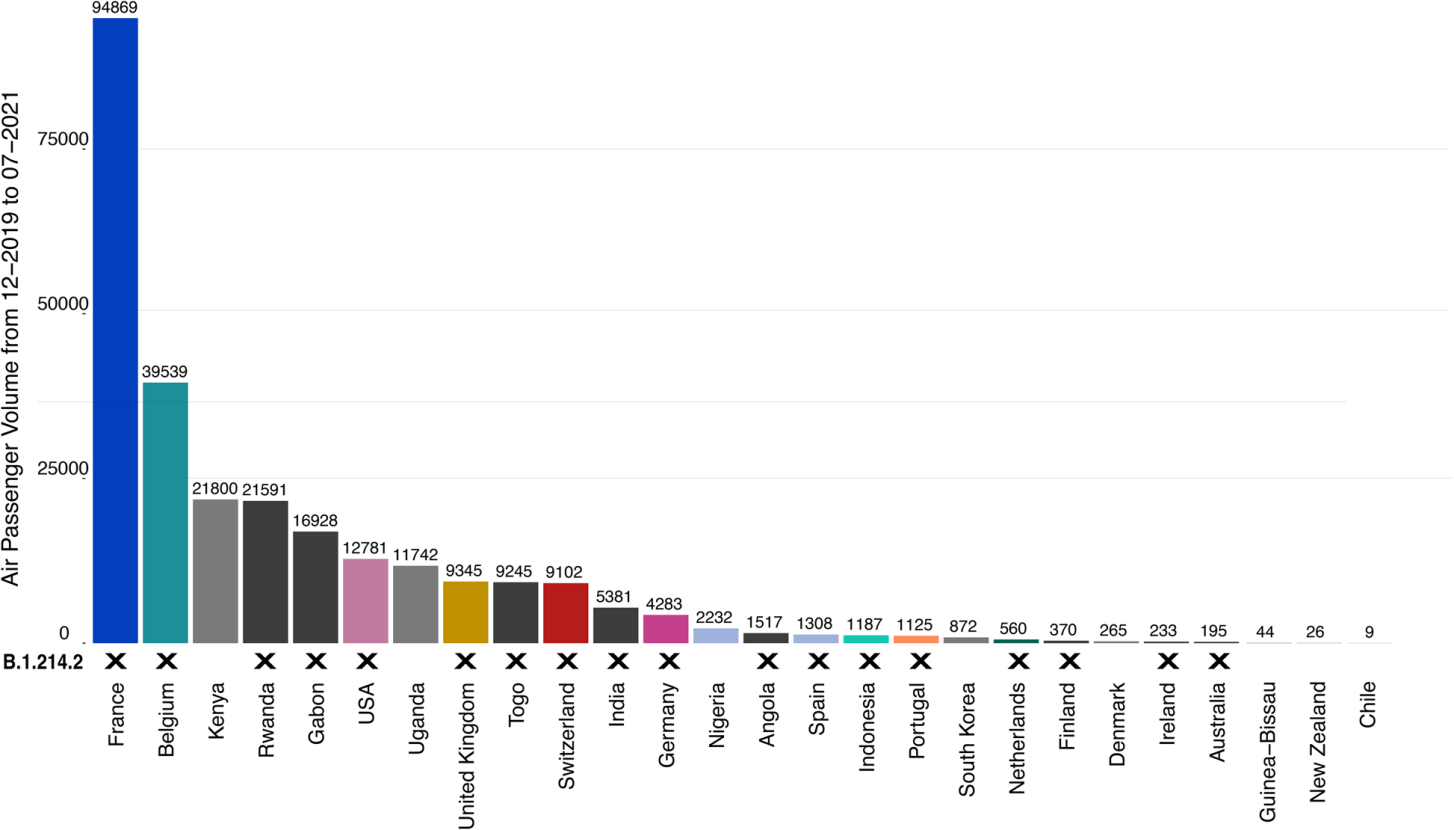
Countries with inbound flights from the Republic of the Congo (RC) or the Democratic Republic of the Congo (DRC) are sorted by total passenger volume between December 2019 and July 2021. RC and DRC are combined since the two airports in their capital cities are used interchangeably by the citizens of both countries. Countries that have submitted at least one B.1.214.2 sequence are marked by ‘X’. Only nine of 27 countries with inbound flights from RC and DRC do not report B.1.214.2 cases. Air passenger data provided by Bluedot [30]

We conducted a travel history-aware Bayesian phylogeographic analysis of the B.1.214.2 subtree using travel data from 14 B.1.214.2 infected patients (Supp. Table 1) using BEAST v1.10.5 [25] (pre_thorney_0.1.2). Our analysis estimated the Republic of the Congo as the likely origin of the B.1.214.2 clade receiving posterior support of 57.5%, followed by France with 29.8% and Belgium with 7.2%. We predicted the tMRCA to mid-June 2020 (2020.443; 95% HPD interval: early Mar 2020 (2020.164) – early September 2020 (2020.681)). We observe two main branches of the B.1.214.2 clade, leading to several independent avenues into Europe (Fig. 5a) originating from the Republic of the Congo. The first likely introduction (88.39% posterior support for the Republic of the Congo) is estimated to have occurred in early August (2020.646, 95% HPD: 2020.490–2020.777) to France and led to the widespread expansion in Europe (Fig. 6b & Supp. Table 2): Belgium in mid-November 2020 (2020.868, 95% HPD: 2020.79–2020.933), Belgium at the end of November 2020 (2020.911, 95% HPD: 2020.853–2020.976) and Switzerland in mid-November 2020 (2020.8454, 95% HPD: 2020.791–2020.888), as well as a reintroduction into the Republic of the Congo in mid-December 2020 (2020.958, 95% HPD: 2020.880–2021.024). The analysis demonstrates a second branch, estimated to originate from the Republic of the Congo origin (99.89% posterior support) seems to lead to four separate Belgian clusters that led to expansion in mostly Belgium, with evidence of transmissions to other Central African and European countries (Fig. 5 and Supp. Table 2). We observe multiple clusters in this second main branch of localized transmissions occuring in France, Germany, Belgium, and the United Kingdom likely from the Republic of the Congo (Fig. 5). These findings strongly suggest the existence of cryptic transmission of the variant in these countries prior to its detection through genomic surveillance in January 2021 (Fig. 1).

The phylogeographic tree provided some context to the high number of cases of the variant in Basel compared to the rest of Switzerland. Out of 230 Swiss sequences, only 5 were from Zürich and 10 from Geneva. Phylogeographic analysis indicates most Swiss B.1.214.2 cases originated from France (or even are French cases sequenced in Switzerland), particularly due to fluidity of the French-Basel border and share evolutionary history with Parisian cases. This suggests a possibly link with the well-traveled train routes between Paris and Swiss border. Contrastingly, German cases were linked to Belgium or the Republic of the Congo and not France or Switzerland.

Our analysis suggests limited involvement of other Central African countries in spreading B.1.214.2 to Europe, possibly due to more sequences from the Republic of the Congo in our dataset (Fig. 1). B.1.214.2 expanded outside Europe and Central Africa only in Indonesia and the United States, likely introduced from Belgium in December 2020 (2020.929, 95% HPD: 2020.838–2021.005) and the United Kingdom (and France before that) in early January 2021 (2021.014, 95% HPD: 2020.961–2021.058), respectively.

To understand the influence of long-distance transition events, we analyzed air passenger data from December 2019 to July 2021, revealing 27 countries that received flights from the Republic of the Congo (RC) or the Democratic Republic of the Congo (DRC). Considering the proximity of their airports, we included both in our analysis. Of these 27 countries, 17 reported B.1.214.2 cases, all with air links to RC or DRC (Figs. 5b and 6). Nine countries with flights from RC or DRC did not report B.1.214.2 cases. However, travel history data indicate potential undetected transmission in three of these, namely Nigeria, Chile, and Guinea-Bissau. This evidence, combined with phylogeographic analysis, suggest the Republic of the Congo as one of the main foci in the dissemination of lineage B.1.214.2

### A large Belgian nursing home outbreak reveals a moderately severe clinical phenotype and a unique B.1.214.2 upper airway immune signature

A B.1.214.2 outbreak in Belgian nursing homes was monitored from January 24, 2021 to March 3, 2021, involving 952 PCR tests on 86 residents and 114 staff, interns, and volunteers. Of these, 54 tested positive, showing higher attack rates in residents (53.5%) than staff (7%). The outbreak, lasting over four weeks, resulted in four deaths among residents aged 85 + , with a case fatality ratio of 8.7%. Whole genome sequencing of 16 high viral load samples confirmed the outbreak was caused by B.1.214.2, with all resident cases clustering together phylogenetically (Fig. 5a).

**Fig. 7:**
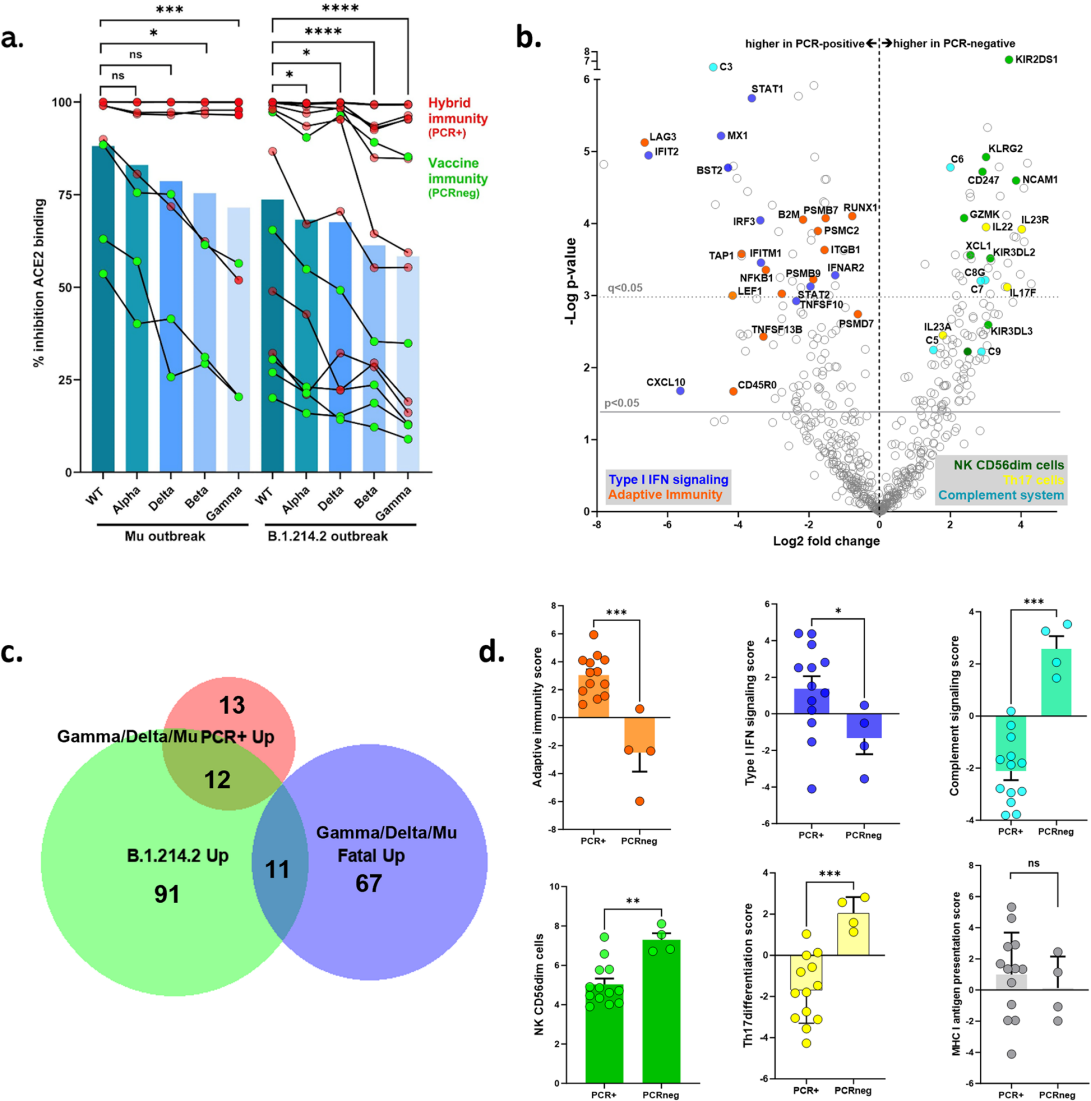
Cross-comparison of large nursing home outbreaks reveals similar systemic neutralizing antibody levels but divergent upper airway immune signature of B.1.214.2 in high-risk elderly. **a** Vaccine-induced (PCR-negative cases) and hybrid (PCR-positive cases) humoral immunity was highly similar in age- and sex-matched residents from B.1.214.2 (*n* = 15) and Mu (*n* = 9) nursing home outbreaks. In both outbreaks, significant decreases in cross-neutralizing antibodies follow the same order of antigenic distance across VOC (WT > Alpha > Delta > Gamma > Beta). Each line represents a single individual, bars represent the median; Kruskal–Wallis test with FDR correction for multiple testing, *p < 0.05, **p < 0.01, ***p < 0.001, ****p < 0.0001. **b** Volcano plot of differentially expressed genes in upper airway of age- and sex-matched B.1.214.2-infected (PCR-positive, *n* = 13) and uninfected (PCR-negative, highly exposed, *n* = 4) nursing home residents. Grey lines show raw *p*-value < 0.05 and FDR-corrected q-value < 0.05 (dotted line). Individual genes belonging to significantly enriched cell types or signaling pathways are highlighted. c. Venn diagram shows a partial overlap (48%, 12/25 genes) in upregulated immune genes shared between B.1.214.2-infected PCR + (“B.1.214 up”) upper airway samples and matched samples of mild/moderate (“Gamma/Delta/Mu PCR + up”) nursing home outbreaks [11]., but only a minor overlap (14%, 11/78 genes) with immune genes upregulated in matched fatal cases (“Gamma/Delta/mu fatal up”), while 91 genes are unique to the B.1.214.2 outbreak. d. Increased Adaptive immunity and type I IFN signaling but decreased NK CD56″dim” cells, Th17 differentiation and complement system in B.1.214.2 PCR + (*n* = 13) vs. PCR-negative highly exposed (*n* = 4) residents. Mann–Whitney test *p < 0.05, **p < 0.01, ***p < 0.001

Despite occurring during the national vaccination campaign, with over 95% of residents vaccinated, infections were recorded post-vaccination without significant differences in case fatality or antibody responses between those infected after the first or second dose. Leveraging our nationwide surveillance effort, neutralizing antibody levels in this outbreak were compared to those in a similar-sized outbreak caused by the Mu variant [11], showing no significant differences in humoral immune response between the two outbreaks., Rather, higher cross-neutralizing antibodies were observed in residents with hybrid immunity (vaccinated and PCR +) as compared to vaccine-induced immunity (PCR-negative) in both outbreaks(Fig. 7A). Significant decreases in cross-neutralizing antibodies follow the same order of antigenic distance across VOC (WT > Alpha > Delta > Gamma > Beta) in both outbreaks (Fig. 7A).

Therefore, we proceeded to compare the global immune response (innate, humoral and cellular) using digital transcriptomics across four independent post-vaccination outbreaks in Belgian nursing homes (B.1.214.2 from this study, and previously described Mu-Gamma-Delta outbreaks [11]). Our strategy was to compare age-, sex- and vaccination status-matched PCR-positive vs. PCR-negative exposed individuals for each separate data set, to obtain immune signatures that represent specific correlates of protection for B.1.214.2 as compared to other variants. A Venn diagram (Fig. 7C) shows a partial overlap (48%, 12/25 genes) in upregulated immune genes shared between B.1.214.2-infected PCR + (“B.1.214 up”) upper airway samples and matched samples of mild/moderate (“Gamma/Delta/Mu PCR + up”) nursing home outbreaks. In contrast, only a minor overlap (14%, 11/78 genes) was found with immune genes upregulated in matched fatal cases (“Gamma/Delta/Mu fatal up”) from previous outbreaks [11]. Of note, 91 out of 114 (80%) upregulated genes were unique to B.1.214.2 upper airway infection (Fig. 7C and Suppl. Table 3). These 91 genes include *IRF3*, which we identified as a “protective” transcriptomic biomarker in previous outbreaks, as well as both type I IFN receptors, *IFNAR1* and *IFNAR2,* the latter was also associated with critical COVID-19 by GWAS (Genome-Wide Association Study) [43]. Our analysis of the immune response to the B.1.214.2 outbreak in nursing homes revealed a distinctive upper airway immune signature, marked by a significant boost in adaptive immunity (Fig. 7B-D, *p* < 0.001). This includes enhanced B-cell and T-cell activity, with increased expression of key signaling and effector genes like *LAG3, TAP1, LEF1, TNFSF13B,* and marker of memory cells *CD45RO*. Additionally, there was a notable rise in innate and antiviral type I interferon (IFN) signaling, highlighted by *IFNAR2/STAT1/STAT2* pathways and antiviral genes such as *MX1, BST2*, and *IFIT2.* Conversely, we observed a reduction in innate Natural Killer (NK) cells and Th17 differentiation, alongside a decrease in complement system genes, without affecting MHC class I antigen presentation. This pattern strongly diverges from previous findings in other nursing home COVID-19 outbreaks [11] and broader cohorts [44–46].

In summary, this cross-comparison underscores a moderately severe clinical impact of B.1.214.2, without significant changes in cross-neutralizing antibodies. The unique upper airway transcriptomic immune signature associated with this variant is characterized by highly specific changes in both adaptive (heightened B- and T-cell activation but lower Th17 signaling) and innate immunity (higher antiviral type I IFN signaling but lower NK and complement system activation).

## Discussion

In this comprehensive study, we present an analysis of the B.1.214.2 variant, encompassing its epidemiological prevalence, mutational profile, immune signature, and origin. Through a travel history-aware phylogeographic analysis, we estimate that the European expansion of B.1.214.2 may have originated from the Republic of the Congo, leading to localized country clusters. We observe several episodes of introduction and reintroduction between European countries and Central Africa, revealing a bi-directional transmission route between the two. The high prevalence of B.1.214.2 in the Republic of the Congo during the same period supports early cryptic transmission previous to European expansion. The strong correlation between B.1.214.2 prevalence and air travel to the Republic of the Congo suggests that air passengers played a major role in the spread of this variant to Europe.

We were originally drawn to the unique nine-nucleotide insertion sequence at the recurrent insertion region (RIR1). This pattern, although novel at the time, has been identified in several other SARS-CoV-2 strains, the most prominent being the Omicron BA.1 sublineage, which has achieved global prevalence [5]. Before Omicron's rise, only 0.3% of all SAR-CoV-2 genomes contained S gene insertions [7], and as seen in our B.1.214.2 analysis, these insertions often bypassed sequencing protocols, which misinterpreted them as artifacts. This bias highlights the need to address sequencing errors to prevent misinformation in large-scale analyses.

Previous phylogenetic work revealed independent tripeptide RIR1 insertions, which suggest convergent evolution at this locus [7]. Many of the variants with this mutation also contain non-synonymous spike substitutions and deletions, hinting at the potential of RIR1 insertions to compensate for deleterious mutations. Gerdol et al., who described and investigated the RIR1 insertion, suggest that the consistent independent emergence of RIR1 insertions in various viral strains, seen in conjunction with Omicron’s emergence, points to the likelihood that RIR1 insertions may offer an evolutionary advantage, although the extent of this advantage remains uncertain [7]. Given these correlations and the predicted impact of RIR1 on the Spike protein's structure, it is plausible that RIR1 could serve a permissive role, potentially offsetting the minor disadvantages of certain non-synonymous spike RBD mutations. This clearly convergent insertion mutation deserves further investigation and shows that surveillance and characterization of non-VOC lineages may help us understand the emergence and advantages of novel pandemic lineages.

The RIR1 in B.1.214.2 is located at R214 and D215, which could be an important locus for variant fitness since the Beta variant (501Y.V2, B.1.351) harbors the mutation D215G. Even though the connection between this loop and neutralizing antibodies remains unclear, there's a prevailing theory that insertions at R214 might counterbalance the effects of relatively harmful mutations in the RBD, such as Q414K and N450K. These mutations may reduce antibody affinity, but they could also lessen the efficiency of protein folding [10].

Our analysis on air passenger data revealed the influence of human migration between continental Europe and Central Africa in the spread of B.1.214.2. The presence of B.1.214.2 cases only in countries with connections to the two Congolese airports suggests a possible pathway for transmission, with three additional potential routes identified through travel history data. The two airports, one in Kinshasa and the other in Brazzaville, are separated by the Congo River and are managed separately. The only five countries with air connections to the Republic of the Congo or the Democratic Republic of the Congo without any evidence of B.1.214.2 in circulation are South Korea, New Zealand, Uganda, Tanzania, and Denmark. Tanzania has reported zero sequences of SARS-CoV-2 due to deprioritization of the pandemic. Uganda, in contrast, has reported 2,031 sequences in total (937 over the B.1.214.2 epidemic period), ten of which are B.1.214 [47–49]. South Korea and New Zealand had very strong travel restrictions and arrival testing protocols during the pandemic, and most likely were able to curb the introduction of B.1.214.2 into their countries. Denmark during this period also had low passenger volume. This could provide some evidence for the success of South Korea and New Zealand in their risk mitigation strategies.

Multiple studies have estimated a Central or Western African origin of SARS-CoV-2 variants, likely due to a combination of factors including importations from Europe, limited early control measures, and ongoing transmission- mostly notably, B.1.620 and A.27 [8, 50, 51]. A recent study explains this trend by arguing that African epidemics are the results of importations from Europe, where early control measures were quickly put in place [52]. In Africa, however, transmission mostly progressed throughout the pandemic with the opportunity for new variants of concern to develop. Although trailing earlier on in the pandemic, several African countries have been able to increase their sequencing capacity, which has enabled phylogeographic studies. This has been greatly in part due to international investment in genomic surveillance, within-Africa collaborations, and progress in reagent and equipment allocation [47]. It is important to mention that, especially due to non-uniform sequencing [23], cases identified in a country do not mean the variant is restricted to that country, but rather that countries doing sequencing function as a window into the region. The Republic of the Congo, with a stronger sequencing capacity, is perhaps a representation of the greater Central Africa region [48, 53]. We suggest further studies from within Central Africa to investigate the transmission of variants in the region.

Alongside sequencing capacity, we relied on travel-history interviews for our origin estimations, making their collection crucial to our results. We have a higher number of documented travel history cases from Belgium, which can be attributed to its robust documentation system and our close collaboration with the hospital systems. Nevertheless, it is important to note that the absence of reported travel history for France, Switzerland, and central African countries does not necessarily mean that such travel cases do not exist. We contacted laboratories in France and Switzerland, aiming to obtain supplemental travel history data that might not be readily available in the GISAID metadata but were unsuccessful due to the strict patient data protections surrounding this information.

Finally, taking advantage of our nationwide surveillance of SARS-CoV-2 nursing home outbreaks, we unveil a moderately severe clinical phenotype in high-risk elderly with an 8.7% case fatality ratio, which is lower than the 20–35% case fatality ratio we observed in other post-vaccine nursing home outbreaks with Gamma, Delta and Mu variants [11]. This clinical phenotype might be explained by systemic broad cross-neutralizing antibodies combined with a novel and distinct upper respiratory tract immune signature in B.1.214.2-infected nursing home residents, as compared to other high-fatality (> 10%) post-vaccine outbreaks with Delta, Gamma and Mu variants. Noteworthy limitations of this study include the absence of SARS-CoV-2 aerosol detection during the outbreak, which we recently demonstrated as a useful marker of long-lasting exposure in other nursing home outbreaks [11]. However, the high attack rate (53.5%), the long duration of the outbreak (> 4 weeks), and the detection of PCR-positive residents on all three floors of the nursing home strongly suggest high exposure in all residents, including the PCR-negatives. Second, no baseline serum samples were available before the outbreak, nor from fatal cases, to compare the levels of pre-existing and/or vaccine-elicited SARS-CoV-2 neutralizing antibodies. A third limitation is the absence of data on staff pandemic preparedness and population incidence of COVID-19 in the surrounding population, which Suñer et al. [54] identified as major predictors of (pre-vaccine) COVID-19 mortality in a large retrospective study of Spanish nursing homes. Major strengths of this study include the comprehensive testing of the complete nursing home, both staff and residents (> 950 PCR tests) for the entire duration of the outbreak, complete metadata, detailed clinical follow-up, and in-depth immunological profiling (systemic anti-S and neutralizing antibodies, upper airway digital transcriptomics). Thus, we found a moderately severe (8.7% case fatality ratio) clinical phenotype of B.1.214.2, with no major difference in cross-neutralizing antibodies across all major VOC (Alpha, Beta, Gamma, Delta) circulating in the same time period. This clinical phenotype of B.1.214.2 was linked to a unique nasopharyngeal immune signature. First, it was characterized by higher adaptive (B- and T-cell mediated) immunity, arguing against immunodedepression or vaccine failure in these infected high-risk elderly, as well as increased type I IFN signaling, also associated with protection from critical COVID-19 [55–57]. Moreover, we also observed lower KIR expression and NK CD56 “dim” cells, as well as lower Th17 and complement activation, which are opposed to our previous findings in similar nursing home outbreaks [11]. Taken together, we propose that this unique upper airway immune signature might explain, at least in part, the peculiar epidemiological history of B.1.214.2, while also reiterating the urgency of a nasal vaccine strategy [58].

## Supplementary Information

Supplementary Material 1.

## Data Availability

The dataset(s) supporting the conclusions of this article are available in the Github repository, https://github.com/amholtz/B12142.

## Data availability

Data is provided within the manuscript, supplementary information files, and github repository.
