# Supplementary material for: Emergence of the B.1.214.2 SARS-CoV-2 lineage with an Omicron-like spike insertion and a unique upper airway immune signature
Source: BMC Infect Dis. 2024 Oct 10;24:1139. doi: 10.1186/s12879-024-09967-w (PMC11468156; doi:10.1186/s12879-024-09967-w)
Supplement: Supplementary file 1 — Supplementary Material 1. [file 12879_2024_9967_MOESM1_ESM.docx]

**Supplemental Figures**


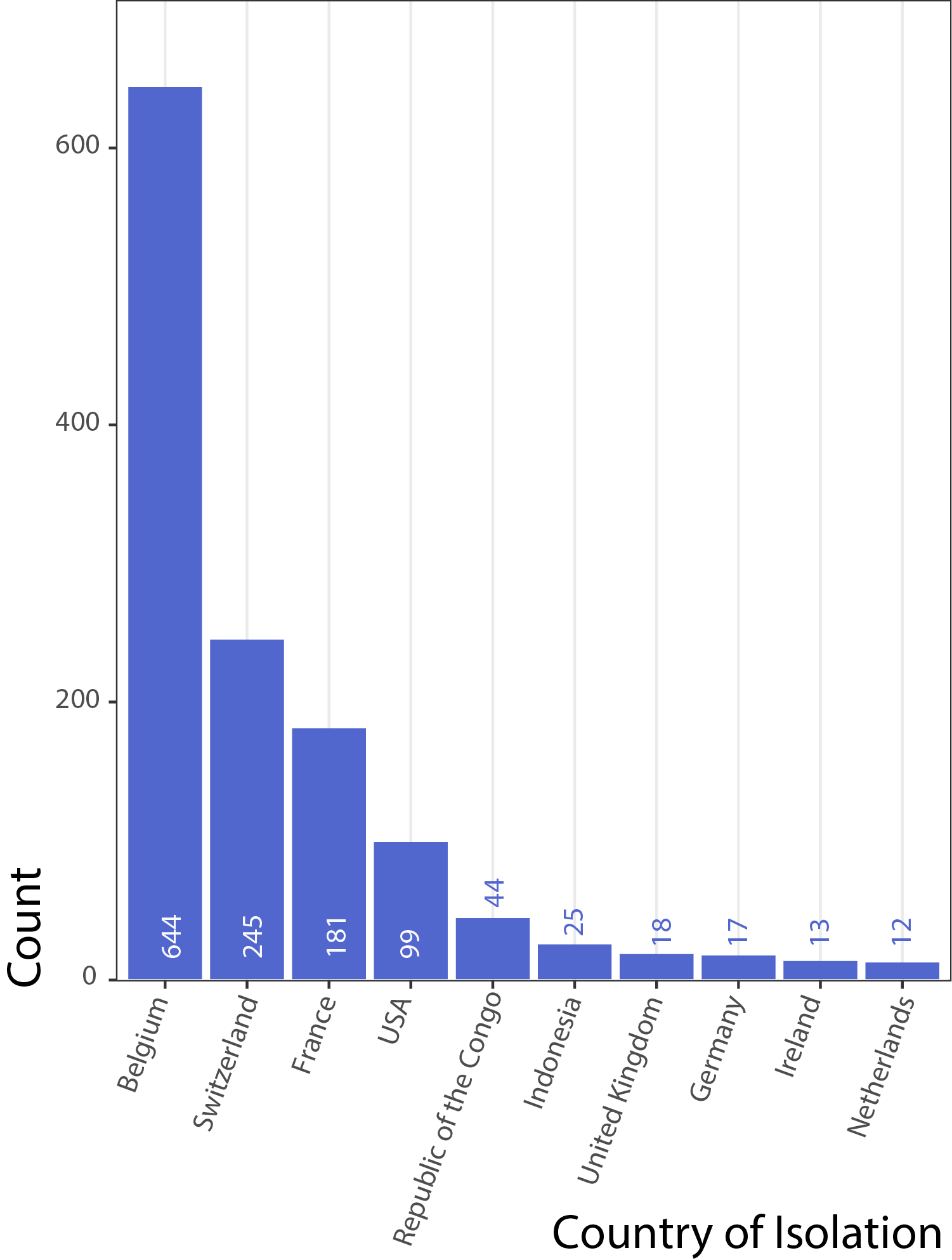

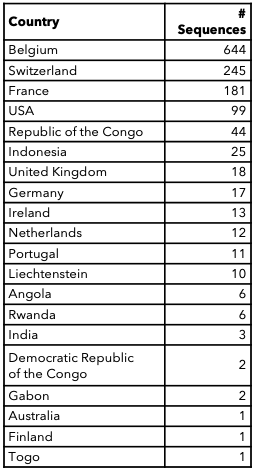


**Supp. Figure 1. Sequenced cases of B.1.214.2 per country.** Number of sequences are shown by country. The ten countries with the most B.1.214.2 sequences are shown in the bar plot on the left. The table on the right shows the total number of countries and number of sequences of the variant submitted to GISAID. This does not include travel-history additions.


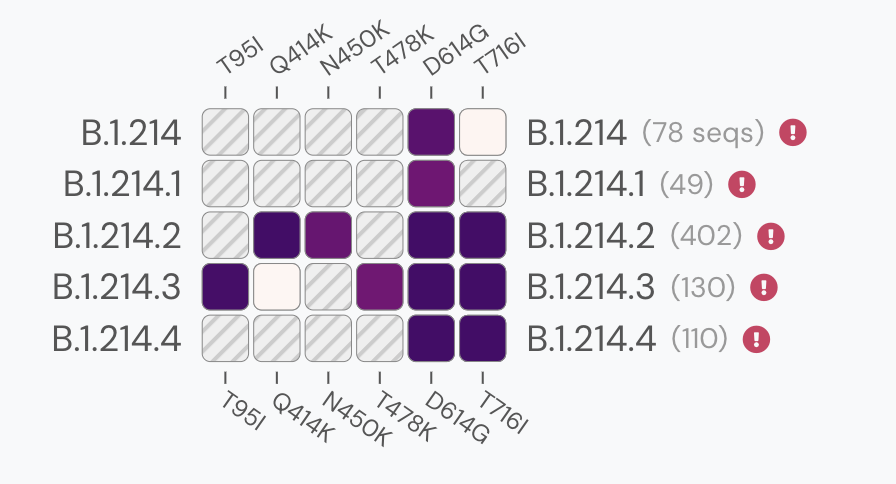


**Supp. Figure 2. Spike protein mutational prevalence across B.1.214 descending lineages.** This figure was taken from outbreak.info

**
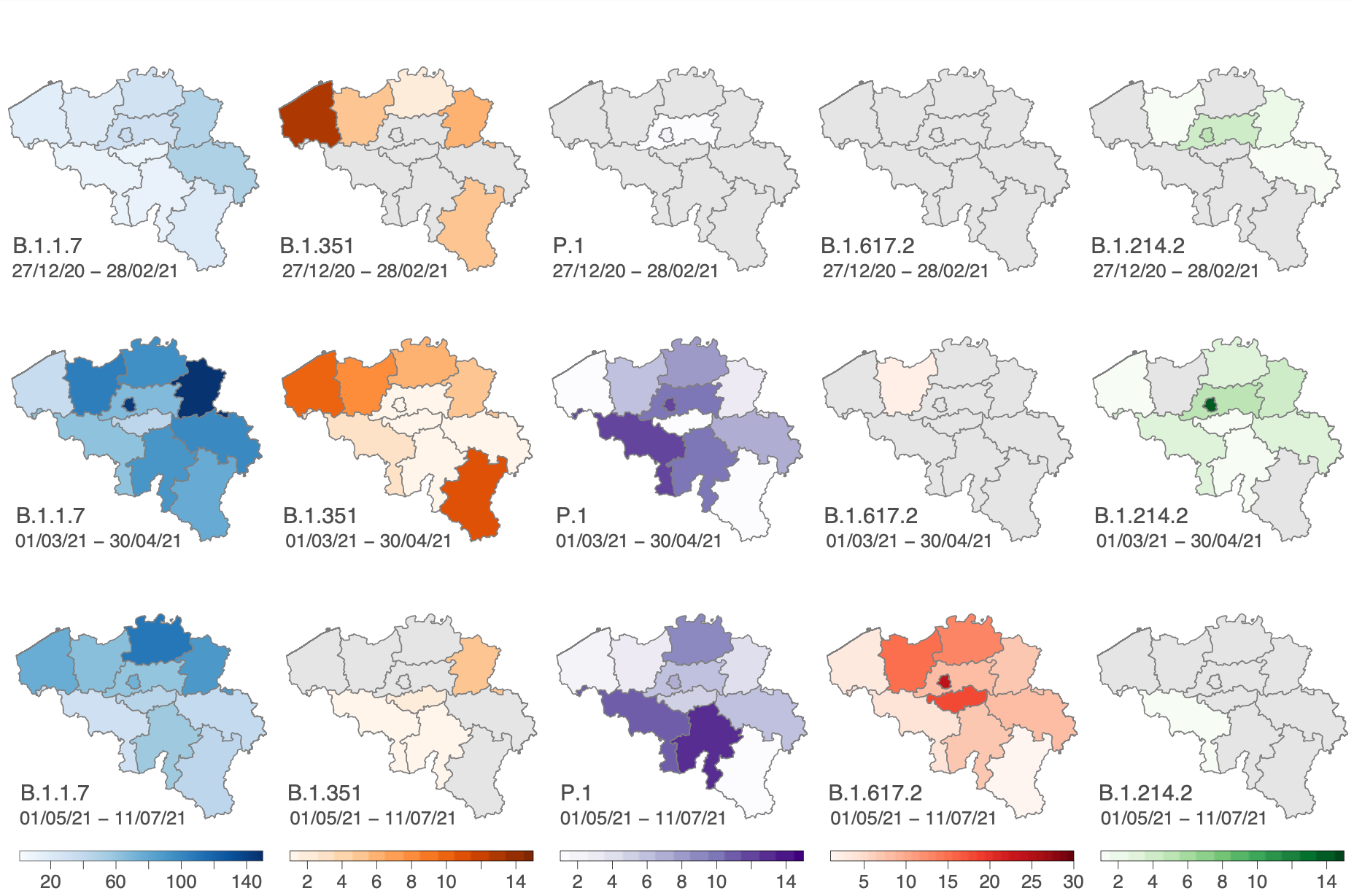
**


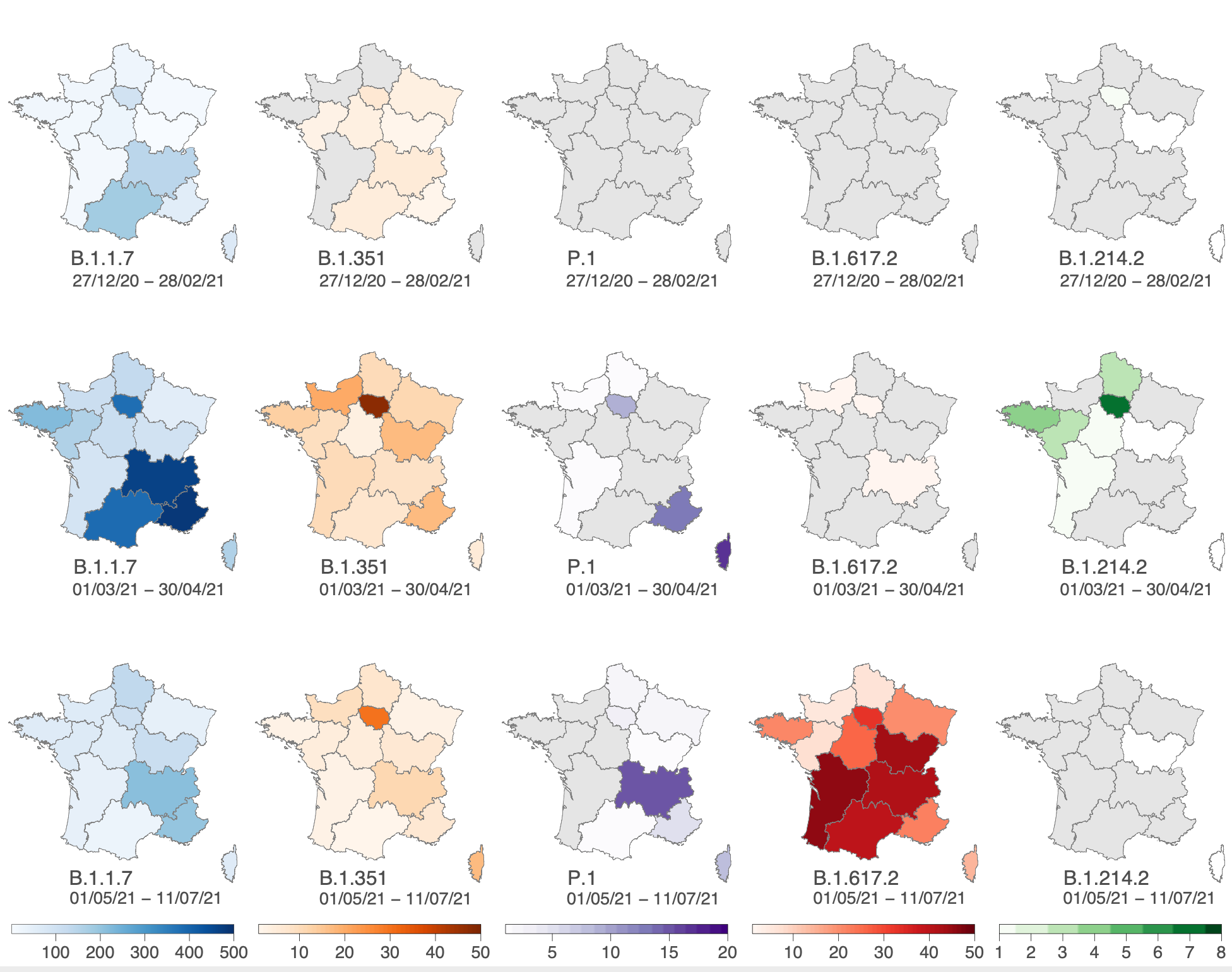


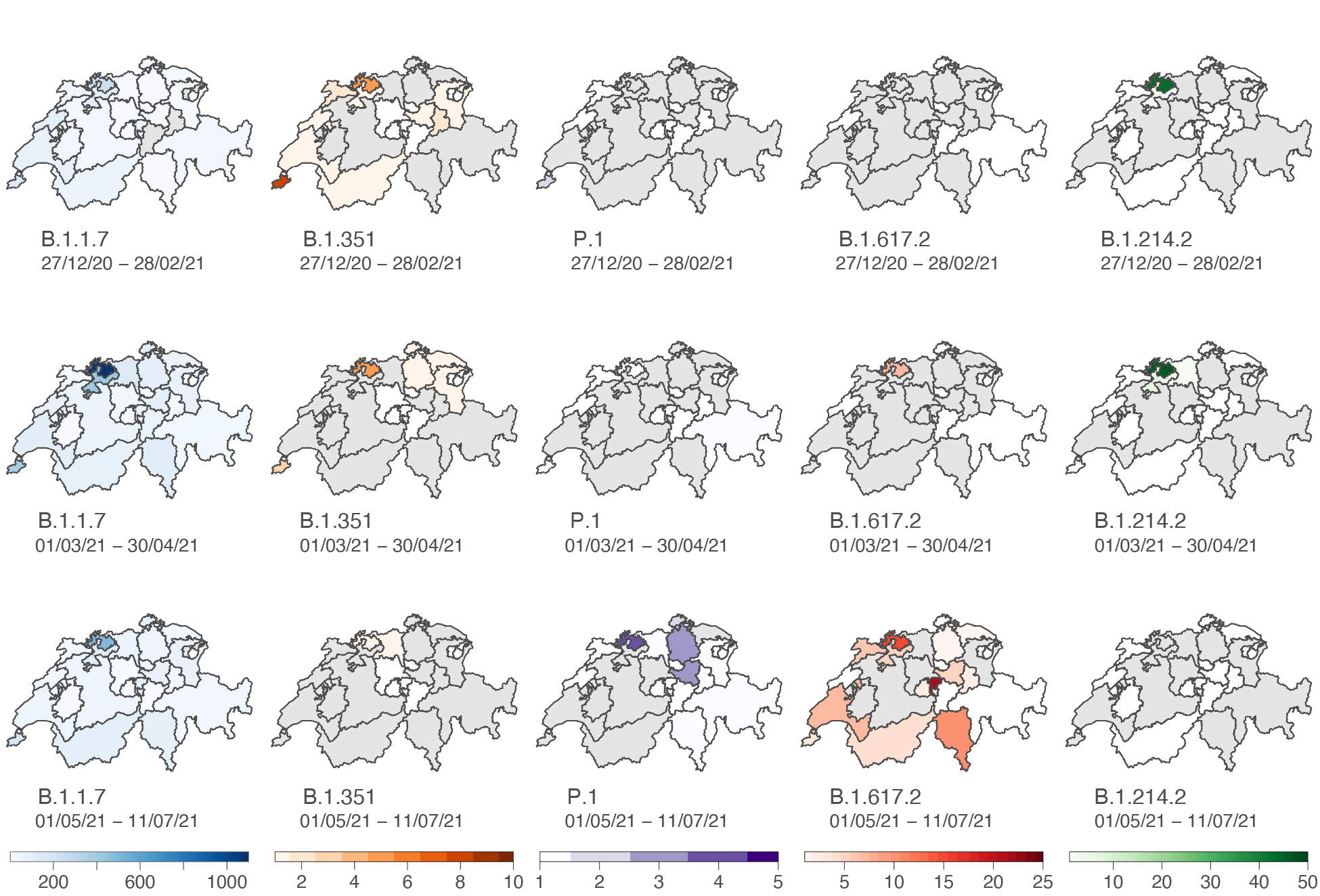


**Supp. Fig. 3: Incidence values of each VOC in Belgian Provinces (cases/10^6^ people), French regions (cases/10^5^ people), and Swiss Canton (cases/10^5^ people) grouped into three successive time periods: 27/12/2021 - 28/02/2021, 01/03/2021- 30/04/2021, 01/05/2021-11/07/2021.** Incidence values are calculated by the number of sequences deposited on GISAID for that region divided by the population of that region/province/canton (2021). Brussels in Belgium, Ile-de-France in France and Basel canton (here merged with Basel-Lanschaft and Basel-Stadt for presentation) in Switzerland show the highest incidence of B.1.214.2. The variant quickly dies off in the last period as B.1.617.2 begins expanding. B.1.214.2 has relatively low incidence values outside of the Basel, Brussels, and Ile-de-France regions.


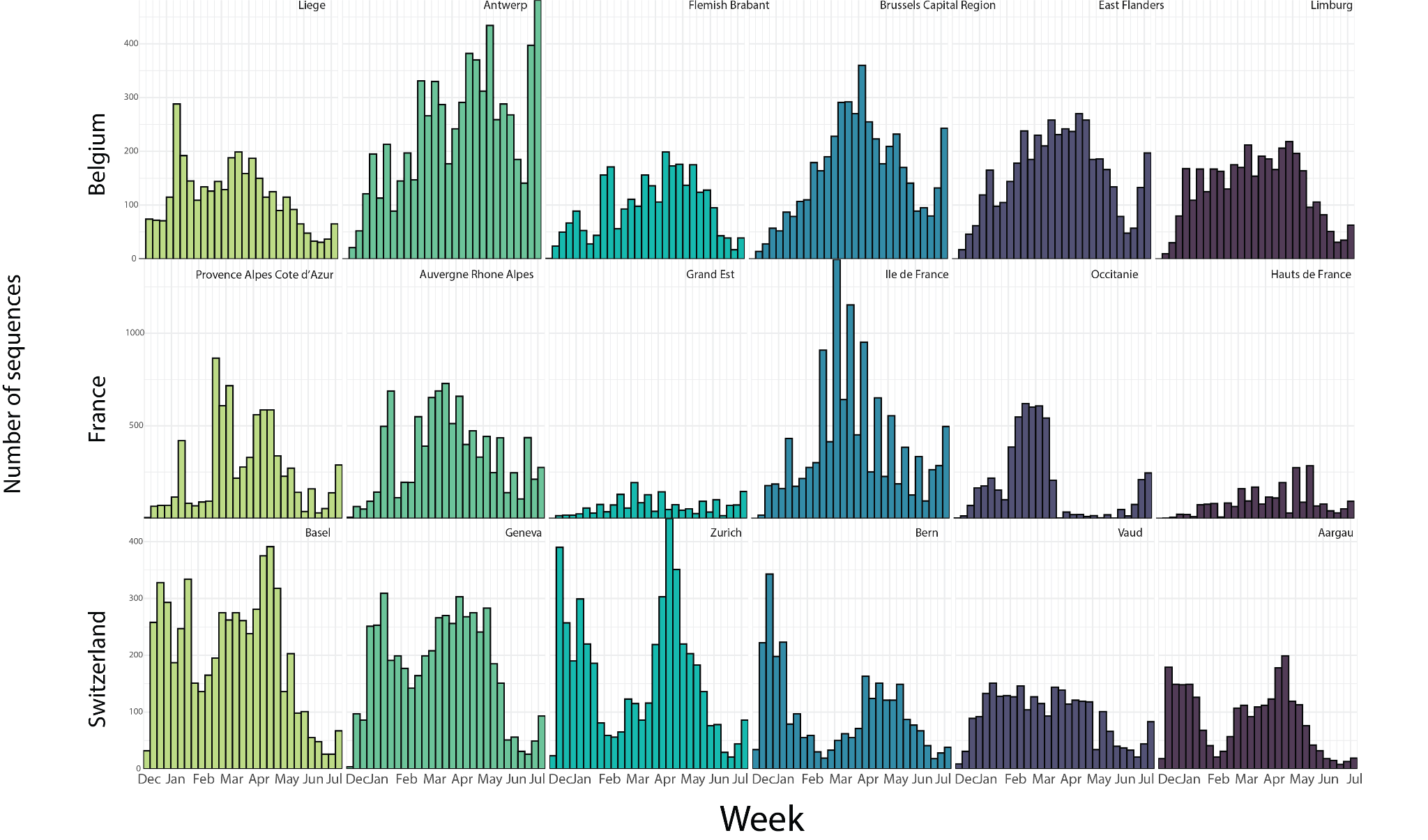


**Supp Figure 4. Number of Sequenced Cases of SARS-CoV-2 in Belgium, France, and Switzerland.** Each country is represented by the sequence totals per week by the six regions with the greatest number of sequences. Sequences submitted to GISAID were grouped by French region, Belgian Province, and Swiss Canton. In Switzerland, Basel is the combination of Basel-Stadt and Basel-Landschaft.

**Supplemental Tables**

**Supp. Table 1. Travel-history associate with B.1.214.2 sequences.** These travel histories were collected from GISAID metadata and from contacting country sequencing laboratories. Travel days are the suspected number of days an individual was in the country of travel before the sample date.

​​

| **Name** | **Location** | **Sampling Date** | **Travel History** | **Travel**  **Days** | **Prior**  **Mean** | **Prior**  **Stdev** |
| --- | --- | --- | --- | --- | --- | --- |
| hCoV-19/Belgium/rega-1632/2021\|EPI_ISL_890291\|2021-01-03 | Belgium | 03/01/2021 | Republic of the Congo | NA | 10 | 3 |
| hCoV-19/Belgium/rega-1638/2021\|EPI_ISL_890294\|2021-01-03 | Belgium | 03/01/2021 | Republic of the Congo | NA | 10 | 3 |
| hCoV-19/Belgium/ULG-11260/2021\|EPI_ISL_833185\|2021-01-07 | Belgium | 07/01/2021 | Republic of the Congo | 3 | NA | NA |
| hCoV-19/Belgium/rega-1778/2021\|EPI_ISL_894200\|2021-01-08 | Belgium | 08/01/2021 | Spain | NA | 10 | 3 |
| hCoV-19/Belgium/rega-1784/2021\|EPI_ISL_894201\|2021-01-08 | Belgium | 08/01/2021 | Spain | NA | 10 | 3 |
| hCoV-19/Belgium/rega-1865/2021\|EPI_ISL_912424\|2021-01-12 | Belgium | 12/01/2021 | Democratic Republic of the Congo | NA | 10 | 3 |
| hCoV-19/Belgium/ULG-12537/2021\|EPI_ISL_1123370\|2021-01-19 | Belgium | 19/01/2021 | Democratic Republic of theCongo | 0 | NA | NA |
| hCoV-19/Belgium/Jessa_55-2105-001118/2021\|EPI_ISL_1128129\|2021-02-04 | Belgium | 04/02/2021 | France | 12 | NA | NA |
| hCoV-19/Belgium/rega-5194/2021\|EPI_ISL_1382699\|2021-03-02 | Belgium | 02/03/2021 | France | NA | 10 | 3 |
| hCoV-19/Belgium/WHT-UMONS-CV2100704647/2021\|EPI_ISL_1524721\|2021-03-09 | Belgium | 09/03/2021 | France | 38 | 10 | 3 |
| hCoV-19/Angola/CERI-KRISP-K014923/2021\|EPI_ISL_2493007\|2021-04-17 | Angola | 17/04/2021 | Guinea-Bissau | NA | 10 | 3 |
| hCoV-19/Angola/CERI-KRISP-K014924/2021\|EPI_ISL_2492994\|2021-04-17 | Angola | 18/04/2021 | Chile | NA | 10 | 3 |
| hCoV-19/Belgium/rega-8462/2021\|EPI_ISL_2833326\|2021-04-27 | Belgium | 27/04/2021 | Germany | NA | 10 | 3 |
| hCoV-19/France/NOR-IPP11606/2021\|EPI_ISL_2259092\|2021-04-30 | France | 30/04/2021 | Nigeria | NA | 10 | 3 |

**Supp. Table 2.** **First country introductions and country transitions up to the root.** First country introductions from the MCC tree are shown here with subtrees larger than or greater to 15 tips. If country introductions are smaller than 15 tips, the two introductions with the largest subtree per country are shown. Country jumps are not nodes, but rather they indicate country change.

|  | **Country Jump** | **Country Jump** | **Country Jump** | **Final Country** | **Tips** | **Date** | **Node Date** | **95% Low** | **95% High** |
| --- | --- | --- | --- | --- | --- | --- | --- | --- | --- |
| Root |  |  |  | RC | 1360 | 10/06/2020 | 2020.443 | 2020.164 | 2020.681 |
| Branch 1 |  |  | RC | France | 827 | 22/08/2020 | 2020.646 | 2020.490 | 2020.777 |
| Branch 1 | RC | RC | France | Switzerland | 247 | 03/11/2020 | 2020.845 | 2020.791 | 2020.888 |
| Branch 1 | RC | RC | France | Belgium | 59 | 13/11/2020 | 2020.868 | 2020.793 | 2020.933 |
| Branch 1 | RC | RC | France | Belgium | 225 | 28/11/2020 | 2020.911 | 2020.853 | 2020.976 |
| Branch 2 |  |  | RC | Belgium | 18 | 29/11/2020 | 2020.913 | 2020.994 | 2020.831 |
| Branch 2 |  |  | RC | Belgium | 219 | 03/12/2020 | 2020.922 | 2020.987 | 2020.854 |
| Branch 1 | RC | France | Belgium | Indonesia | 25 | 03/12/2020 | 2020.929 | 2020.838 | 2021.005 |
| Branch 1 |  | RC | France | UK | 86 | 09/12/2020 | 2020.940 | 2020.885 | 2020.984 |
| Branch 1 |  | RC | France | RC | 7 | 16/12/2020 | 2020.958 | 2020.880 | 2021.024 |
| Branch 2 |  |  | RC | UK | 23 | 16/12/2020 | 2020.959 | 2020.915 | 2020.992 |
| Branch 2 |  |  | RC | France | 20 | 26/12/2020 | 2020.986 | 2020.917 | 2021.045 |
| Branch 1 | RC | France | Switzerland | Portugal | 6 | 28/12/2020 | 2020.992 | 2020.944 | 2021.030 |
| Branch 1 | RC | France | Belgium | Ireland | 8 | 29/12/2020 | 2020.994 | 2020.943 | 2021.028 |
| Branch 2 |  |  | RC | Germany | 8 | 03/01/2021 | 2021.005 | 2020.914 | 2021.076 |
| Branch 2 |  |  | RC | Belgium | 118 | 05/01/2021 | 2021.010 | 2021.010 | 2021.008 |
| Branch 1 | RC | France | UK | USA | 80 | 06/01/2021 | 2021.014 | 2020.961 | 2021.058 |
| Branch 2 |  |  | RC | Belgium | 41 | 12/01/2021 | 2021.031 | 2021.035 | 2020.997 |
| Branch 2 |  | RC | Belgium | France | 20 | 02/02/2021 | 2021.088 | 2021.042 | 2021.133 |
| Branch 2 |  | RC | Belgium | Netherlands | 4 | 15/02/2021 | 2021.124 | 2021.094 | 2021.145 |
| Branch 2 |  | RC | Belgium | Germany | 10 | 17/02/2021 | 2021.130 | 2021.099 | 2021.161 |
| Branch 1 |  | RC | France | Ireland | 3 | 25/02/2021 | 2021.149 | 2021.108 | 2021.178 |
| Branch 1 |  | RC | France | Portugal | 4 | 01/04/2021 | 2021.248 | 2021.228 | 2021.265 |
| Branch 2 |  | RC | Belgium | Netherlands | 3 | 29/05/2021 | 2021.406 | 2021.371 | 2021.428 |

**Supp. Table 3: Overlap between upregulated genes in B.1.214.2 infected nursing home residents and Gamma/Delta/Mu-infected nursing home residents with mild/moderate vs. fatal outcome**

| **Gene Set** | **Num. of genes** | **Gene Symbol** |
| --- | --- | --- |
| B.1.214 up PCR+ up | 12 | CXCL10, SERPING1, CCL5, FYN, GZMB, TAP1, BST2, LAG3, CIITA, LILRB1, CCL2, IFIT2 |
| B.1.214 up Fatal up | 11 | IFI35, TP53, NOS2, UBE2L3, CDKN1A, ILF3, IL6ST, ATG5, CCL7, CTNNB1, PSMC2 |
| PCR+ up | 13 | PRF1, ZAP70, CTLA4_all, C1S, C1R, CARD9, EBI3, KLRC3, PDCD1, CXCL11, CD2, IRF8, LAIR1 |
| B.1.214 up | 91 | FKBP5, LGALS3, IFI16, CLEC7A, TOLLIP, LAMP3, CD45R0, TNFSF10, TNFSF13B, CEACAM1, MUC1, ARG2, LEF1, GBP1, CUL9, STAT2, IDO1, IFNAR2, CD46, IRF5, MALT1, ITGAE, TMEM173, CTSS, TNF, MX1, TRAF4, PSMB8, C1QBP, RAF1, CX3CL1, IKZF2, MYD88, IFITM1, HLA-DRB1, CASP2, CXCL9, ITGB1, IRF3, CD40, CD59, CXCL13, PPBP, CFH, HLA-DMB, CLEC5A, MRC1, KIT, IRAK2, CFB, PSMB5, CXCL1, JAK2, CHUK, IL10, TBK1, CASP1, STAT3, ATG7, MAPK1, CCL20, MCL1, RELA, PSMB7, SOCS1, IL13RA1, CCND3, STAT1, IFIH1, CFD, SMAD3, IFNAR1, RUNX1, IKBKAP, TRAF5, TCF4, B2M, PSMD7, IRAK3, TAP2, NFKB1, CD58, CD164, AHR, DUSP4, CASP3, PSMB9, NOTCH2, FAS, C3, ITGA4 |
| Fatal up | 67 | TRAF2, XCL1, KCNJ2, ITGAM, IFNG, CD79B, IL17F, C14orf166, TNFAIP6, C8G, IL26, BID, C4A/B, IFNA1/13, LILRA4, CR2, IFNB1, TNFSF15, TLR2, CCL16, TIGIT, TGFB1, RAG2, EOMES, CCL3, BCAP31, CD19, ABL1, B3GAT1, PDGFB, CCR1, ITGA2B, NCAM1, THY1, PTPN22, IL6R, CDH5, CCR5, CD99, TLR1, CTSG, IL28A, LILRA5, IL17B, CD80, IL22, RORC, ICAM5, CD1A, KIR3DL2, ARG1, STAT5A, CCL19, C6, PTGER4, KIR3DL3, SRC, FCAR, NT5E, C9, PLA2G2A, CD79A, CSF2, CCRL2, AIRE, CCL13, KLRC1 |
